# High-density Mapping of a Posteroseptal Accessory Pathway Using Open-window Mapping

**DOI:** 10.19102/icrm.2021.120102S

**Published:** 2021-01-15

**Authors:** Daniel R. Frisch

**Affiliations:** ^1^Jefferson Heart Institute, Philadelphia, PA, USA

**Keywords:** Ablation, accessory pathway, Advisor HD Grid, high-density mapping, open-window mapping

A 21-year-old man with a family history of Wolff–Parkinson–White syndrome and a personal history of chest pain and palpitations was referred for ablation. The baseline electrocardiogram (ECG) showed sinus rhythm at 62 bpm with a P–R interval of 80 ms, a QRS duration of 130 ms, and a corrected QT interval of 422 ms. There was a delta wave that was positive in leads I, II, and aVL; isoelectric in leads III and aVF; and negative in V1 but positive in V2 **(Figure 1)**.

High-density, three-dimensional mapping was performed across the posteroseptal aspect of the tricuspid valve during sinus rhythm using the Advisor™ HD Grid Mapping Catheter, Sensor Enabled™ and the EnSite Precision™ electroanatomic mapping system. We employed the open-window mapping (OWM) strategy described by Schricker et al.^1^ OWM relies on the maximum absolute dV/dt value from each bipolar signal on the high-density grid to collect activation points in patients with accessory pathway conduction. The “open window” does not distinguish atrial, pathway, and ventricular signals from one another and mapping leads to visualization of the channel of activation across the mapping segment (in this case, the tricuspid valve). We localized the precise pathway location within the right inferoparaseptal (posteroseptal) region **(Video 1)**.

Ablation was performed with the TactiCath™ DF, Sensor Enabled™ catheter set at a flow of 17 cc/minute, power of 25 W, and a goal contact force of 8 to 40 g, delivered for 10 to 30 seconds per lesion. Aided by high-density mapping, the pathway was eliminated successfully. In follow-up, the patient has remained symptom-free with no evidence of preexcitation on subsequent ECGs.

## Figures and Tables

**Figure 1:**
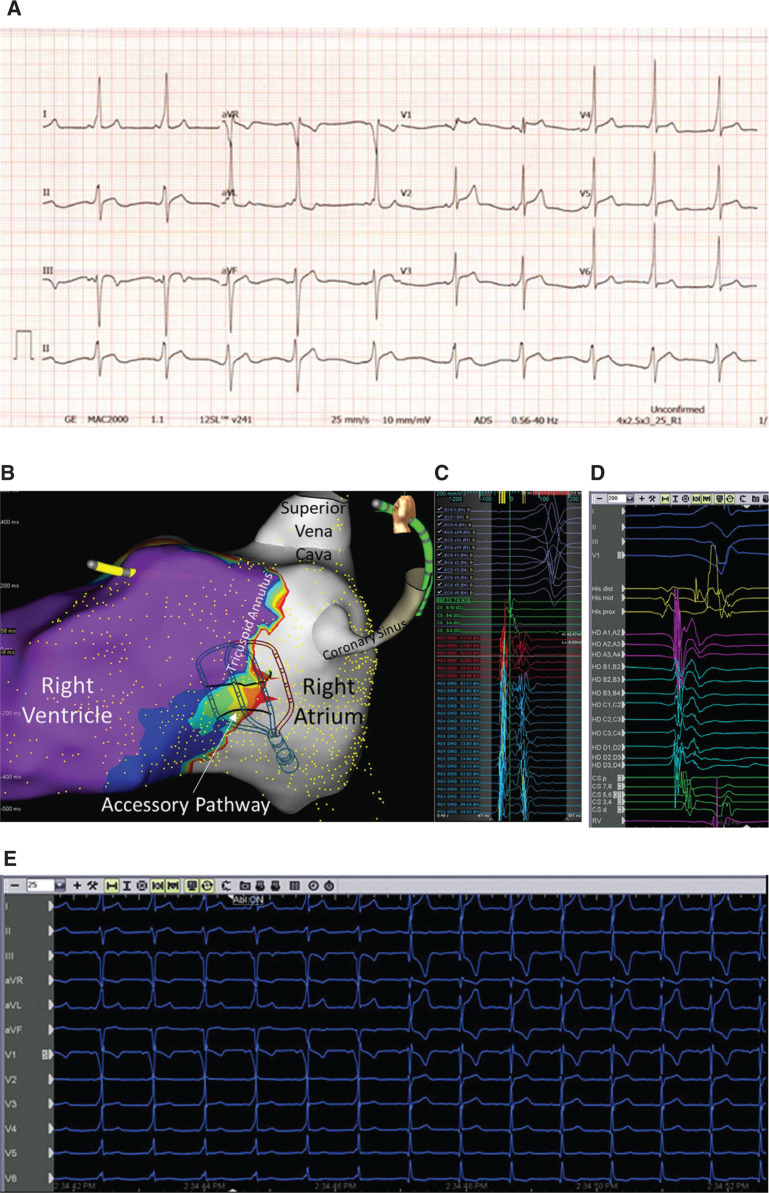
The value of high-density mapping using an OWM strategy. **A:** Preablation ECG showing preexcitation. **B:** High-density map of the right atrium, tricuspid annulus, and right ventricle using the Advisor™ HD Grid catheter to localize the posteroseptal location of the accessory pathway. **C:** High-density grid signals at the accessory pathway (AP) site recorded with the EnSite Precision™ electroanatomic mapping system. **D:** High-density grid signals at the AP site recorded from the GE CardioLab™ (GE Healthcare, Chicago, IL, USA) recording system. **E:** ECG during ablation showing elimination of the AP.

**Figure 1:**
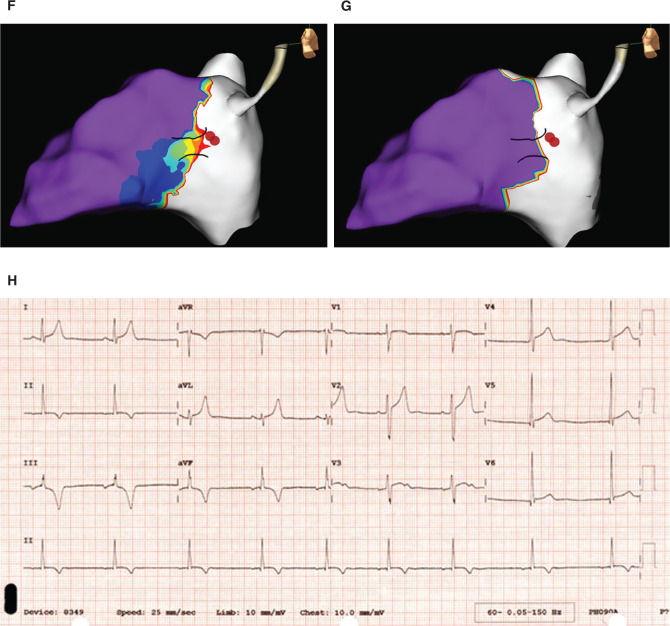
**F:** Activation map showing the location of the AP and successful lesion sites prior to ablation, while there is still AP conduction. **G:** Activation map showing the location of the AP and successful lesion sites after ablation when there is no longer AP conduction. **H:** Postablation ECG showing no preexcitation.

**Video 1.** Video showing propagation through the pathway prior to ablation. The accessory pathway is outlined in black. The ablation lesions are shown in red.
